# Analysis by LC-MS/MS of polar pesticides in fruits and vegetables using new hybrid stationary phase

**DOI:** 10.1016/j.mex.2021.101306

**Published:** 2021-03-17

**Authors:** Víctor Cutillas, Amadeo R. Fernández-Alba

**Affiliations:** University of Almería, Department of Chemistry and Physics, Agrifood Campus of International Excellence (ceiA3), Ctra. Sacramento s/n, La Cañada de San Urbano, Almería 04120, Spain

**Keywords:** Pesticides, Liquid chromatography, Hilic, Ion exchange, Mass spectrometry

## Abstract

Highly polar pesticides are frequently used in agriculture. However, their physicochemical properties make very difficult the analysis of these compounds following common procedures. Polar pesticides show poor retention and peak shapes in the common stationary phases used for multiresidue methods of pesticides. For this reason, multiple columns with different stationary phases have been developed to perform the analysis of these particular compounds. The column evaluated in this method uses a new hybrid stationary phase with a mixed-mode between hydrophilic interaction liquid chromatography (HILIC) and ion exchange interactions. The retention modes are dependant on mobile phase conditions and can be easily switched. The aim of this study can be summarized in the next bullet points:

• Performance evaluation of 10 anionic compounds in different column sizes of the hybrid stationary phase.

• Validation of the method in terms of sensitivity, linearity, and matrix effects in four different matrices: tomato, orange, onion and quince.

• Improvement of the retention time robustness

## Graphical abstract

**Figure:**
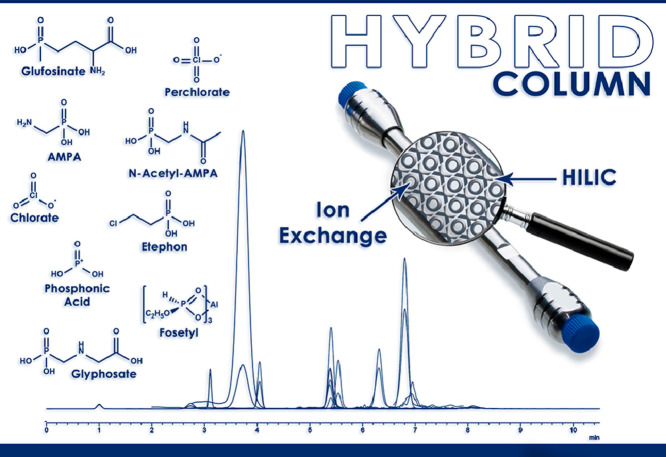

**Table:** Specifications Table

Subject area	Chemistry
More specific subject area	Analytical Chemistry (Pesticide residue analysis)
Method name	Methanol extraction of polar pesticides without acidification
Name and reference of original method	QuPPe
Resource availability	https://www.eurl-pesticides.eu/docs/public/tmplt_article.asp?CntID=887&LabID=200&Lang=EN

## Methods details

### Chemicals and reagents

The pesticide standards used for the optimization and subsequent validation were provided by LGC (Teddington, United Kingdom) and Sigma-Aldrich (Steinheim, Germany) and were stored at 30 °C. A standard-mix solution was prepared using individual stock solutions. Individual stock solutions (800–1000 mg L^−1^) were prepared from each standard in water and methanol and stored in the dark at 30 °C in amber glass vials. Acetonitrile and methanol (LC-MS quality) were obtained from Fluka Analytical (Steinheim, Germany). LC-MS grade water from Fisher Chemical (Fair Lawn, NJ, USA) was used. Formic acid was purchased from Sigma Aldrich (Steinheim, Germany). The salts employed in the extraction method (anhydrous magnesium sulfate, sodium chloride, sodium hydrogenocitrate sesquihydrate, and sodium citrate tribasic dihydrate) were supplied by Sigma-Aldrich (Steinheim, Germany).

### Sample treatment

The four matrices studied (tomato, orange, onion, and quince) were obtained from different local markets in Almería (Spain). An analysis of the samples was performed to ensure that they did not contain any of the polar pesticides studied, and those samples were selected as a blank. The extraction procedure was according to the following protocol: A 10  g portion of the homogenized sample was weighed into a 50  mL PTFE centrifuge tube. Next, 10  mL methanol and an appropriate volume of water was added [1]. The addition of water to our samples was as follows: 0.5  mL were added to the tomato matrix portion, 1 mL to the onion one, and 1.5 mL was added to quince and orange matrices portions. In each extraction, 50  µL of 10  mg/L ^13^C^15^N glyphosate was added. The samples were shaken in an automatic axial extractor (AGYTAX®; Cirta Lab S.L.,Madrid, Spain) for 4  min. The samples were then centrifuged(3500  rpm) for 5  min. The extracts were stored in PTFE tubes. To evaluate peak area repeatability, matrix effects, and linearity, blank extracts were spiked with the mixture pesticides at the desired concentration.

### LC-MS/MS analysis

The LC analysis was performed using a Nexera UC (Shimadzu Corporation, Kyoto, Japan). The LC separations were carried out on a Raptor Polar column (2.7 µm, 50 × 2.1  mm) from Restek (Bellefonte,PA,USA). An EXP Direct Connect Holder equipped with a guard column cartridge (2.7 µm, 5 × 2.1  mm) was connected to the column, both acquired from Restek (Bellefonte,PA,USA). This column can switch between polar retention modes by modifications in mobile phase conditions. When using a high percentage of an organic mobile phase like acetonitrile, the stationary phase uses the HILIC retention mechanism. On the other hand, when the percentage of water increases, ion-exchange is the dominant retention mode. Mobile phase A was water, whereas mobile phase B was acetonitrile, both mobile phases contained 0.5% formic acid. The percentages of formic acid tested were: 0, 0.1, 0.3, 0.5, and 1. If the percentage of formic acid in the mobile phase is lower than 0.5%, peak shape performance decrease, especially in the glyphosate case. The oven temperature was set at 35 °C. The total flow used was kept constant at 0.6 mL min-1. The mobile phase gradient started with 35% of mobile phase A. It is increased to 90% at minute 2 and then, the flow was kept isocratically during 7 min. Mobile phase decrease from this minute to minute 10 where it reaches again 35% and is maintained for 2 min for re-equilibration.

The LC chromatograph is coupled to a triple quadrupole mass spectrometer 8060 (Shimadzu Corporation, Kyoto, Japan). The study was carried out employing an electrospray ionization source (ESI). The interface temperature was set at 300  °C. The desolvation line (DL) was set at 200 and 400 °C in the case of the heat block. The interface voltage used was 3 kV. Regarding nebulizer, heating, and drying gas flows: 3 L min-1, 10 L min-1, and 10 L min-1 were used, respectively. All the pesticides evaluated including the internal standard (^13^C^15^N glyphosate) were manually optimized by using precursor ion search. The identification criteria applied are described in the SANTE document [2]. Acquisition windows of  ±  0.5 min were established for each pesticide. All the parameters related to the optimization of the compounds can be checked in Table 1.

**Table 1:** Mass spectrometer parameters of the anionic compounds included in the method.

Compound	RT (min)	precursor ion (m/z)	CE1	SRM 1	CE2	SRM 2
AMPA	1.023	110.1	23	79	22	63
Ethephon	6.881	143.3/145.3	11	107	11	107
Fosetyl	9.734	109.1	14	81	23	63
Glufosinate	2.662	180.1	33	63	18	85.1
Glyphosate	5.782	167.9	20	63	14	79
Glyphosate C13N15	5.766	171	25	63	37	79
N-acetyl-AMPA	6.492	152.1	27	63	17	110.1
Phosphonic acid	9.833	81.1	25	79	13	63
Chlorate	6.867	83.1	21	67	31	51
Perchlorate	5.835	99.2	21	83.1	37	67

RT: retention time; CE: collision energy.

### Column selection

**Fig. 1:**
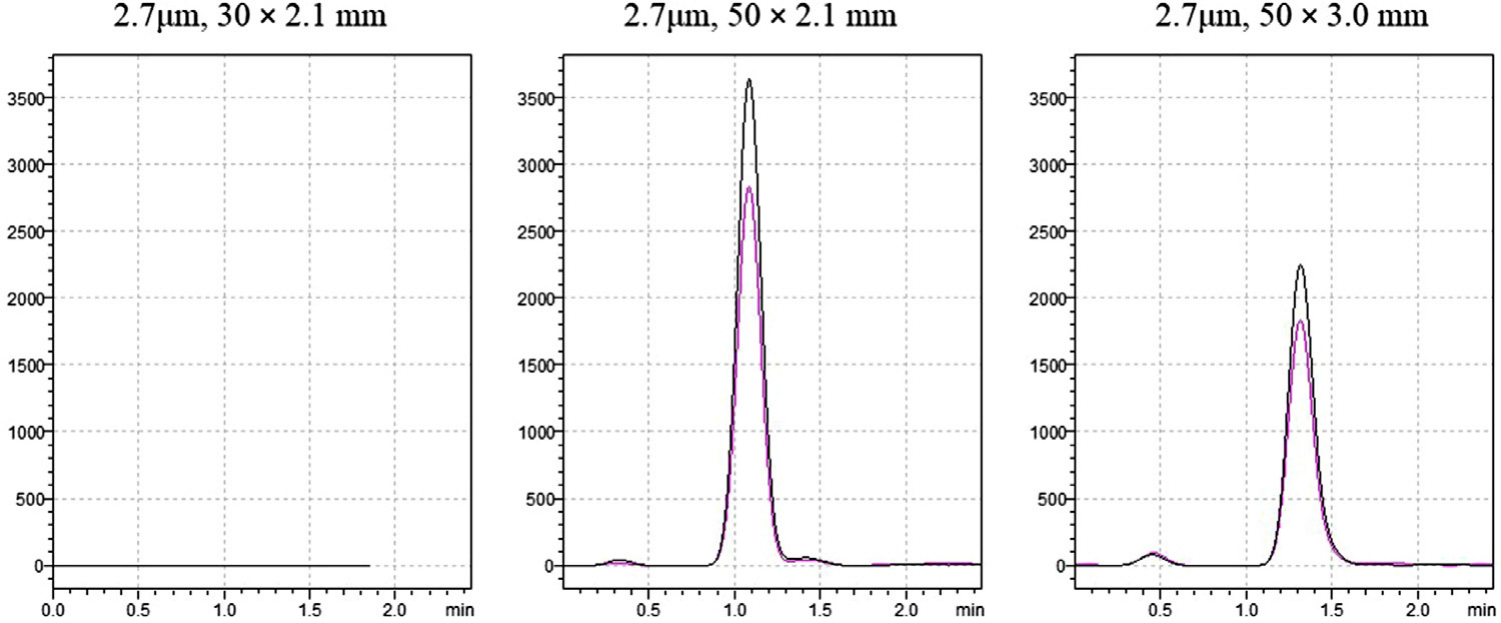
Chromatograms of 50 µg/Kg of AMPA in orange matrix using three different columns dimensions.

The stationary phase evaluated in this study was initially checked in three different column sizes. The specifications of the first column tested were: 2.7 µm of particle size, 30 mm length, and 2.1  mm of internal diameter. All the compounds can be detected except aminomethyl phosphonic acid (AMPA). This compound elutes at the very beginning of the chromatogram (0.8 min) and despite it can be detected in some matrices like tomato or onion, no peak was detected in orange and quince matrices. Using 50mm column length this problem disappears and AMPA can be detected in all the matrices. Two internal diameters (ID) of this 50mm column were tested: 2.1 and 3.0 mm. To obtain similar peak shapes between both ID is necessary to increase the mobile phase flow to 0.8  mL/min when 3.0  mm ID column was used. Moreover, the sensitivity using the 2.1 mm column was slightly better as can be observed in Fig.1. Taking into account these facts, the 2.7 particle size, 50 mm length and 2.1  mm ID was the column selected for the study.

### Method validation

Instrumental limits of quantification (LOQ), linear range, and matrix effects were the parameters evaluated during the validation of the method (Table 2). Matrix-matched calibration curves at 5 different concentration levels (10,20,50,100 and 500 µg/Kg) were injected in the four matrices studied (tomato, onion, quince, and orange). No dilution was applied to the vials, the composition was the same as the extracts: water/methanol (1:1). The compound's LOQ range between 10 and 50 µg/Kg. However, in all the cases, the LOQ levels meet the requirements of the EU maximum residue levels (MRL) for each matrix. Regarding linearity, weighted linear regression (1/x) was used. The response was considered linear if individual residuals deviated <20%. The compounds fulfill the residuals requirements with the exception of chlorate in tomato matrix and perchlorate in all the matrices. For these particular cases, there is a loss of linearity above 100 µg/Kg. The matrix effects were calculated through the comparison of the slopes of the matrix-matched calibration curves injected with the one injected in pure solvent. A value between 0 and 20% is considered a non-existent matrix effect. Signal deviations between 20 and 50% are considered low or medium matrix effects. However, strong matrix effects are considered when the value is above 50%. All the results indicate suppression of the signal except for N-Acetyl-AMPA and chlorate in quince matrix. Orange was the matrix providing higher matrix effects in all the pesticides. AMPA was the compound more affected by the matrix effects, being close or above 50% in the four matrices.

**Table 2:** Validation results of the polar compounds evaluated in the four matrices studied.

	Tomato			Onion			Quince			Orange		
	LOQ	Linear Range	M.E.	LOQ	Linear Range	M.E.	LOQ	Linear Range	M.E.	LOQ	Linear Range	M.E.
**AMPA**	10	10-500	-45%	20	20-500	-56%	20	20-500	-66%	50	50-500	-80%
**Ethephon**	20	20-500	-4%	50	50-500	-22%	50	50-500	-20%	50	50-500	-34%
**Fosetyl**	10	10-500	-2%	10	10-500	-5%	10	10-500	-5%	10	10-500	-12%
**Glufosinate**	10	10-500	-15%	10	10-500	-16%	10	10-500	-23%	10	10-500	-40%
**Glyphosate**	20	20-500	-12%	10	10-500	-9%	20	20-500	-18%	10	10-500	-20%
**N-acetyl-AMPA**	20	20-500	-34%	20	20-500	-23%	10	10-500	11%	20	20-500	-16%
**Phosphonic acid**	50	50-500	-11%	50	50-500	-8%	50	50-500	-24%	50	50-500	-20%
**Chlorate**	20	50-500	-7%	10	10-500	-24%	10	10-100	25%	20	20-500	-30%
**Perchlorate**	10	10-100	-52%	10	10-100	-37%	10	10-100	-11%	10	10-100	-69%

LOQ: limit of quantification; M.E.: Matrix effects

### Robustness

The repeatability of the method was evaluated through the injections of matrix-matched vials 30 times in a row. The area reproducibilities were all below 20%, however, there was a decrease in the retention time of the compounds in every injection. This decrease was not very noticeable in AMPA and glufosinate, but the impact in the rest of the compounds after thirty injections was between 0.1–0.3 min. This variation suffers a slight increase as columns of higher length were used. This issue can be solved by changing the guard column after 30–40 injections approximately. However, to increase the working life of the guard columns different backflushing methods were tested. After numerous flows (0.4–1.2 mL/min), solvents (acetonitrile and water), and additives (0.5–1% formic acid), the backflushing using 1% of formic acid in water for 60  min was the combination that provides better results. As can be observed in Table 3, there is a partial recovery of the retention time for the majority of the compounds analyzed except in the quince matrix.

**Table 3:** Retention time modification after backflushing the guard column.

	Onion	Tomato	Orange	Quince
	RT after backflushing	RT after backflushing	RT after backflushing	RT after backflushing
**AMPA**	-0.011	-0.006	+0.004	-0.003
**Ethephon**	-0.007	+0.026	-0.004	-0.025
**Fosetyl**	+0.015	+0.041	+0.018	-0.026
**Glufosinate**	0	+0.003	+0.017	+0.002
**Glyphosate**	+0.008	+0.069	+0.069	+0.001
**N-acetyl-AMPA**	+0.017	+0.063	+0.051	+0.006
**Phosphonic acid**	-0.043	+0.064	+0.046	-0.056
**Chlorate**	+0.001	+0.011	-0.062	-0.048
**Perchlorate**	+0.009	+0.03	-0.056	-0.042

RT: Retention time.

**Fig. 2:**
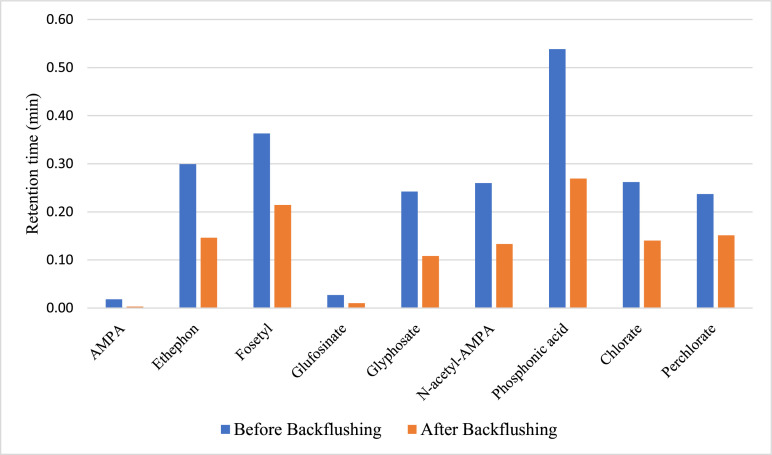
Retention time decrease after 30 injections of the same vial in quince matrix.

Nevertheless, there was another positive effect after this backflushing. There was a reduction in the retention time decrease during the same amount of injections. This fact can be observed in Fig. 2. In quince matrix, despite there was not any increment of the initial retention time after the backflushing, there was a remarkable reduction of the retention time decrease. This situation is common to all the matrices except for glyphosate, N-acetyl-AMPA, and phosphonic acid in tomato matrix and AMPA, and phosphonic acid in onion matrix.

## Declaration of Competing Interest

The authors declare that they have no known competing financial interests or personal relationships that could have appeared to influence the work reported in this paper.
